# North London Hospital for Consumption

**Published:** 1892-06-04

**Authors:** 


### NORTH LONDON HOSPITAL FOR CONSUMPTION.

The festival dinner in aid of the funds of this institution was held at the Hotel Metropole on the 28th ult., Mr. Henry Irving in the chair. A certain amount of freshness and novelty was imparted to the proceedings by the fact that the guests were not altogether of the class which one usually meets at hospital dinners. For, besides Members of Parliament, medical men, County Councillors, and philanthropists, who are always present on such occasions, there were to be seen representatives of art, literature, music, play-writing, and all branches of that profession of which the Chairman is so eminent a member.

Mr. Henry Irving, who was most warmly received, in the course of an excellent speech on behalf of the institution, said that the North London Consumption Hospital made a stronger appeal to their common humanity at the present time than it ever did before. A year ago the world was startled by the news of a great discovery; it was thought that that fell disease, consumption, was baffled at last; that a German physician had found the precious means of arresting it, at least in its earliest stages, and snatching the sufferer from the brink of the grave. No one could ever forget the joy which this seeming messenger of deliverance bore on its wings over both hemispheres. (Hear, hear.) Alas, rejoicing was premature, and the talisman against a cruel malady had yet to be charmed by science out of the secrets of Nature. But this very disappointment ought to give new zest to their efforts to provide every solace, every palliative, everything that medical skill had mastered for the relief of their fellow-creatures, who, in the midst of a dire misfortune, had been robbed of so great a hope. (Hear, hear.) He knew nothing in human history more pathetic than this story of Dr. Koch's failure, a failure which all must pray might prove to be the prelude of a triumph which would spread more beneficence throughout the earth than had ever been achieved by the arts of government. (Applause.)

"How small of all that human hearts endure,
That part which kings or laws can cause or cure."

But, in the meantime they knew their duty. They could not avert disease, but they could try to mitigate it and assuage its pains. (Hear, hear.) They could aid those who struggled through darkness into light, and assisted beneficent nature in restoring shattered strength, and they might help to lessen the pain of the sufferers by at least making their surroundings less terrible. (Hear, hear.) The North London Hospital for Consumption was an institution which was the need of a great city, for to the great city came many whose own poorer and less highly organised localities could not afford the opportunities for special treatment. The North London Hospital was thought of and wrought for in no mean or local spirit. It stood "four-square to all the winds that blow," and on each and every side was a portal open to the afflicted. (Applause.) It was so strong in its purpose that it violated with intention even the Scriptural canon regarding the choice of a site. True, it was a house set on a hill, for so it lifted its head high over the London fog and damp, and so gave life and vigour to those poor souls to whom to breathe the damp air was death—but it was not built on a rock. If they would find a fault with the organisers of this great charity, they might do so for this, that they had chosen to build their house on sand. For this monument of the piety of the rich and poor alike sprang from that island of Bagshot sand, which lifted itself above the wilderness of London clay. (Laughter.) But he could assure them that though the hospital was built on sand, the foundations were all right. (Applause.) They had a firmer base than any earth could yield, for they rose from the mercy, the pity, and the gratitude of human hearts when they were nearest God. (Applause.)

The contributions received as a result of the dinner were announced by the able and energetic Secretary (Mr. Lionel F. Hill), as having reached the respectable total of £2,296, towards which the Chairman had collected £755, and the Chairman of the Committee of the hospital (Mr. B. A. Lyon), £750.

